# Physiological Function of Mycobacterial mtFabD, an Essential Malonyl-CoA:AcpM Transacylase of Type 2 Fatty Acid Synthase FASII, in Yeast *mct1*Δ Cells

**DOI:** 10.1155/2009/836172

**Published:** 2009-10-21

**Authors:** Aner Gurvitz

**Affiliations:** Section of Physiology of Lipid Metabolism, Center for Physiology, Pathophysiology and Immunology, Institute of Physiology, Medical University of Vienna, Schwarzspanierstrasse 17, 1090 Vienna, Austria

## Abstract

*Mycobacterium tuberculosis* mtFabD is an essential malonyl-CoA:AcpM transacylase and is important for vital protein-protein interactions within type 2 fatty acid synthase FASII. mtFabD contacts KasA, KasB, FabH, InhA, and possibly also HadAB, HadBC, and FabG1/MabA. Disruption of mtFabD's interactions during FASII has been proposed for drug development. Here, the gene for a mitochondrially targeted mtFabD was ectopically expressed in *Saccharomyces cerevisiae mct1*Δ mutant cells lacking the corresponding mitochondrial malonyl-CoA transferase Mct1p, allowing the mutants to recover their abilities to respire on glycerol and synthesize lipoic acid. Hence, mtFabD could physiologically function in an environment lacking holo-AcpM or other native interaction partners.

## 1. Introduction

A new approach for eradicating tuberculosis has been proposed that centers on perturbing vital protein-protein interactions associated with the essential mycobacterial process of type 2 fatty acid synthase, FASII [1]. FASII is dedicated to the production of mycolic acids and is targeted by isoniazid, which represents only one of less than a handful of veteran first-line drugs for combating *Mycobacterium tuberculosis*. Mycolic acids are very long chain *α*-branched *β*-hydroxylated fatty acids that help form the pathogen's defensive layer [2].

A critical step in mycobacterial FASII is represented by the transfer of the malonyl moiety from coenzyme A (CoA) to acyl carrier protein (activated holo-AcpM), which is effected by a malonyl-CoA:AcpM transacylase mtFabD (Rv2243), so as to generate malonyl-AcpM [3]. Importantly, mtFabD also plays a key role in holding together FASII enzymes within a protein complex [1]. The structure of this complex is presumed to occur as a constellation of a core triad, with each one of the three identical core units independently contacting a single condensing enzyme, KasA, KasB, or FabH. KasB is further linked to Pks13 responsible for subsequent maturation of the nascent mycolic acid, and both KasA and KasB provide condensing activities for additional methyltransferases during this process [1].

The FASII core unit [1] is thought to be composed of mtFabD, FabG1/MabA (representing 3-oxoacyl-AcpM reductase), InhA (2-*trans*-enoyl-AcpM reductase), and 3-hydroxyacyl-AcpM dehydratase, ostensibly HadAB and HadBC [4]. Within this core, mtFabD occurs in close proximity to—and also forms specific protein-protein interactions with—itself, InhA, and possibly also with HadAB and HadBC. In addition, it can also be directed to contact mutant versions of nondimerizing FabG1/MabA [1]. It was proposed that disrupting mtFabD interactions may be detrimental for mycobacterial survival, and this should be considered as a possible new approach in the search for novel antituberculous drugs [1]. Hence, it would be important to study mtFabD function in a situation where these formal protein-protein interactions are altered or nonexistent, such as in yeast mitochondrial FASII.

**Figure 1:**
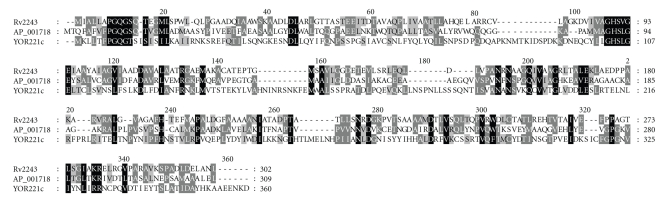
Comparison of *M. tuberculosis* mtFabD with its *E. coli* and *S. cerevisiae* homologs. Multalin- and Genedoc-based comparison of the deduced amino acid sequence of *M. tuberculosis* FabD (Rv2243), *E. coli* FabD (AP_001718), and *S. cerevisiae* Mct1p (Yor221cp). Dashes indicate the arrangement of the sequences for best fit. Black shadings refer to conserved amino acid residues among all three sequences whereas the darker and lighter grey shadings denote regions with more relaxed residue similarities not necessarily shared by the full set of sequences.

The fungal FASII equivalent of mtFabD is exemplified by *Saccharomyces cerevisiae* Mct1p [5]. Mct1p is structurally more distant to the mycobacterial protein than is *Escherichia coli* FabD (Figure 1), whose latter temperature-dependent inactivation can be compensated for by ectopically expressing mtFabD [3]. Yeast mutant cells devoid of Mct1p contain underdeveloped mitochondria, fail to respire or produce sufficient levels of lipoic acid, and are exclusively fermentative [5]. Here, mutant yeast cells expressing a mitochondrially targeted form of mycobacterial mtFabD were compared to an otherwise isogenic wild-type strain for growth, respiration, and lipoic acid production, and the implications are discussed.

## 2. Materials and Methods

The *fabD* sequence was amplified from H37Rv genomic DNA by thermocycling using oligonucleotides MLS-FabD F 5′-TTATCCATGGTTGCGTTGCTCGCACCCGGAC-3′ and FabD R 5′-TATTAAGCTTATAGGTTTGCCAGCTCGTCC-3′ that introduced 5′*Nco*I and 3′*Hin*dIII sites. The amplified *fabD* DNA was processed in such a way that it was preceded by the nucleotides for the Coq3p [6] mitochondrial leader sequence (MLS), and the gene fusion was ligated behind the *CTA1* promoter, as described [7]. The final mtFabD expression plasmid, referred to as pYE352:CTA1-COQ3-FABD, was based on a *URA3*-marked YEp352 multicopy plasmid [8]. A control *URA3*-marked multicopy plasmid vector YEplac195 [9] was used to transform the wild type or the otherwise isogenic mutant to uracil prototrophy. Nucleotide sequencing of the *fabD* insert verified that no mutations were introduced during the amplification process and that the *COQ3-fabD* junction remained intact.

The wild-type yeast strain BY4741 (*MATa his3*Δ*1leu2*Δ*0met15*Δ*0ura3*Δ*0*) and its *mct1*Δ derivative (*yor221c::kanMX*) were obtained from EUROSCARF (http://www.uni-frankfurt.de/). Transformation of yeast strains was performed using a published method [10], and transformants were selected on solid synthetic defined-glucose medium lacking uracil (SD-Ura) that consisted of 0.67% (wt/vol) yeast nitrogen base without amino acids, 2% (wt/vol) D-glucose, and 3% (wt/vol) agar, with all supplements added except for uracil (Sigma-Aldrich Inc. St. Louis, MO). Synthetic complete glycerol medium (SCglycerol) was made up essentially as above, but with the addition of uracil and the replacement of glucose with 3% (wt/vol) glycerol as the sole carbon source. Other standard yeast [11] and *E. coli* [12] media used are described. Respiration competence was assayed by overlaying cells grown on solid SD-Ura medium with 0.1% (wt/vol) 2,3,5-triphenyltetrazolium chloride (TTC) in 0.067 M phosphate-buffered saline and 1.5% (wt/vol) low-melting temperature agarose [13]. Lipoic acid content of yeast strains was monitored by a biological assay described previously [14, 15] using the lipoic acid deficient *E. coli* strain JRG33. Multalin (http://npsa-pbil.ibcp.fr/cgi-bin/npsa_automat.pl?page=/NPSA/npsa_multalin.html) and Genedoc (http://www.nrbsc.org/gfx/genedoc/index.html) were accessed to construct the amino acid alignment in Figure 1.

## 3. Results

**Figure 2:**
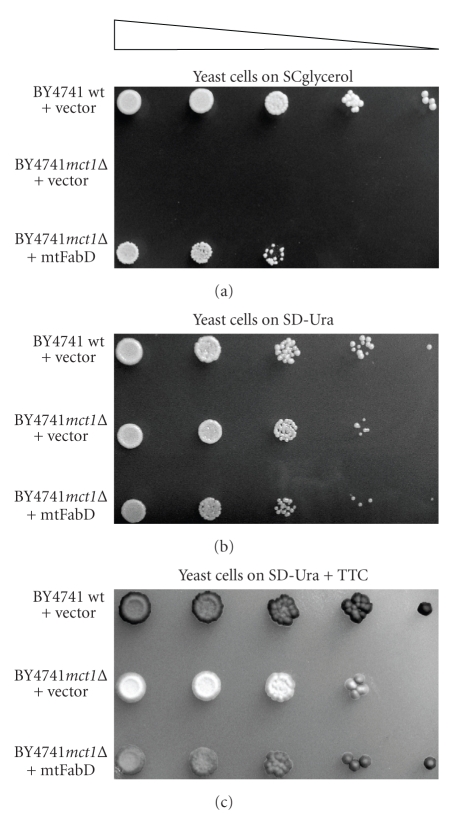
Respiratory growth of *S. cerevisiae* *mct1*Δ mutant cells expressing *M. tuberculosis* mtFabD. Wild-type yeast cells harboring a YEplac195 plasmid vector and *mct1*Δ cells similarly transformed to uracil prototrophy or expressing mitochondrially targeted Rv2243 (mtFabD) from a YEp352 plasmid (both plasmid types are marked with *URA3*) were grown on SD-Ura medium that selected for plasmid presence, and following serial dilution (triangle), were applied to solid media that consisted of (a) 3% (wt/vol) glycerol (SCglycerol) or (b) 2% (wt/vol) glucose (SD-Ura). The plates were incubated at 30°C until single colonies appeared, and recorded photographically. For (c), following an additional 4 d incubation, the SD-Ura plate was overlaid with 0.1% (wt/vol) 2,4,5-triphenyltetrazolium chloride (TTC), and the development of the red chromophore was monitored.

To examine whether mtFabD would be able to act as a physiological malonyl-CoA transferase in *S. cerevisiae*, the corresponding gene was expressed in yeast *mct1*Δ cells devoid of the native mitochondrial malonyl-CoA:Acp1p transferase Mct1p. The mycobacterial protein was generated as a hybrid construct that was preceded by the yeast Coq3p MLS capable of directing proteins to the mitochondria [6]. As controls, *mct1*Δ cells and the corresponding wild type were transformed with a *URA3*-marked YEplac195 plasmid vector [9]. Thereafter, the three strains were propagated overnight on SD-Ura glucose medium selecting for plasmid presence. Following tenfold serial dilution, cultures were spotted onto solid SD-Ura or SCglycerol media, and the plates were incubated at 30°C until single colonies were detectable. The results demonstrated that *mct1*Δ mutants expressing mtFabD resembled to an extent the wild-type strain in that they were capable of some growth on glycerol as the sole carbon source (Figure 2a). As anticipated, those mutant cells harboring a *URA3*-marked plasmid vector that was devoid of a sequence for transferase activity were not able to grow or divide on this medium, albeit they demonstrated ample fermentative growth on glucose (Figure 2b). To investigate whether the growth observed for mtFabD-expressing mutant cells correlated with a regenerated electron transfer chain, 2,4,5-triphenyltetrazolium chloride (TTC) was applied to the previous SD-Ura plate. The results demonstrated that mutant *mct1*Δ cells expressing mtFabD were similar to the wild type in that they generated the red chromophore (Figure 2c). Hence, mtFabD effected the recuperation of the electron transfer chain in mutant *mct1*Δ cells, which enabled them to resume respiratory growth on a nonfermentable carbon source.

Finally, to place mtFabD action within the context of fatty acid biosynthesis in yeast mitochondria, lipoic acid production was examined in the three strains. In this test, lipoic acid was prepared from triplicate yeast cultures grown on SD-Ura, and the extracts were used to supplement a synthetic growth medium into which were inoculated auxotrophic bacterial cells. The values were calculated based on a standard curve that plotted bacterial growths versus known amounts of lipoic acid that had been added to the otherwise restrictive medium. The results showed that extracts derived from the wild-type strain led to a level of bacterial growth that was equivalent to 208 ± 67 ng lipoic acid per gram wet weight of yeast cells (values are mean ± S.D., *n* = 3), whereas those derived from *mct1*Δ mutant cells harboring the plasmid vector supported only a nominal growth commensurate with 20 ng lipoic acid per gram wet weight. Importantly, extracts from mutant cells expressing mtFabD yielded near wild-type bacterial growth levels of 184 ± 37 ng lipoic acid per gram wet weight. Taken together, ectopically expressed mtFabD could compensate for the loss of fungal Mct1p, and the significance of this result is discussed.

## 4. Discussion

*Mycobacterium tuberculosis* causes widespread human death and immense suffering, and it is estimated that about 2 million people die annually from tuberculosis. No less than one third of humanity is thought to be burdened with *M. tuberculosis*, which kills more adults than any other disease due to a single infectious agent [2]. Numerous tuberculosis patients are now infected with resistant strains, and so the development of novel therapeutics against *M. tuberculosis* is a pressing issue. Recently, a new approach for battling tuberculosis has been proposed, in which mtFabD-dependent protein-protein interactions considered pivotal for the production of mycolic acids by FASII are to be singled out for perturbance [1].

In this study, *M. tuberculosis fabD* was shown to be interchangeable with the *S. cerevisiae* gene for Mct1p during respiratory growth and *de novo* lipoic acid biosynthesis. The chemistry underlying the present phenotype rescue of a yeast FASII mutant is fundamentally different compared to previous demonstrations of physiological function within this context, for example, using InhA [7], HadAB and HadBC [16], or FabG1/MabA and FabG4 [17]. This difference lies in the fact that here mtFabD modified the thioester bond to the 4′-phosphopantetheine group of yeast acyl carrier protein Acp1p, whereas the previously tested enzymes acted on Acp1p's cargo acyl group. There are a number of surprises associated with the present finding. One, yeast cells were capable of metabolizing very GC-rich genetic information (67.3% for *fabD* compared with only 37.6% for *MCT1*) and were successful in expressing a mitochondrially targeted heterologous protein that could fold into its native conformation in a wholly nonmycobacterial environment. Two, despite mtFabD's marked preference for mycobacterial AcpM over bacterial ACP [3], it nevertheless tolerated the evolutionarily more distant yeast Acp1p. And three, although mtFabD interacts within its native environment with additional FASII enzymes [1, 18], the mycobacterial transacylase was still functional in the absence of these interaction partners.

It should be underscored that the existence *per se* of an FASII complex in mycobacteria is not disputed as a result of this study, nor are the contacts made by mtFabD with its partners. Nevertheless, the recent demonstration of FabG1/MabA function in fungi [17] has put into question the extent to which protein contacts are really important for mycobacterial FASII [18]. The present finding that mtFabD can also work in yeast should therefore raise the alarm for those subscribing to the point of view that disrupting FASII-dependent associations would be lethal to *M. tuberculosis* and perhaps dampen some of their expectations that compounds capable of annulling these contacts would have pharmacological uses. The work outlined here additionally underscores the versatility of this fungal system for exploring the function of pathogen proteins in complete quarantine from their native metabolic networks.
